# Optimizing Thyroglobulin Interpretation to Reduce Ultrasound Examinations in the Follow‐Up of Differentiated Thyroid Carcinoma

**DOI:** 10.1111/cen.70029

**Published:** 2025-09-02

**Authors:** Luca Giovanella, Petra Petranović Ovčariček

**Affiliations:** ^1^ Department of Nuclear Medicine and Thyroid Center Gruppo Ospedaliero Moncucco, Via Soldino Lugano Switzerland; ^2^ Clinic for Nuclear Medicine University Hospital of Zürich Zürich Switzerland; ^3^ Department of Oncology and Nuclear Medicine University Hospital Center Sestre Milosrdnice Zagreb Croatia; ^4^ School of Medicine University of Zagreb Zagreb Croatia

**Keywords:** clinical decision limits, differentiated thyroid carcinoma, follow‐up, immunoassay variability, receiver operating curve analysis, Thyroglobulin, ultrasound

Dear Editor,

We read with great interest the recent article by Seo et al. [1], which presents a retrospective analysis of patients with low to high‐risk differentiated thyroid carcinoma (DTC) who underwent thyroidectomy, with or without adjuvant or ablative radioiodine therapy, between 2010 and 2023. These patients subsequently underwent fine‐needle aspiration cytology (FNAC) for ultrasound (US)‐suspicious findings. The study categorised pre‐FNAC serum thyroglobulin (Tg) and anti‐Tg antibody (TgAb) levels into three groups: negative (Tg < 0.2 µg/L and negative TgAb), positive (Tg ≥ 0.2 µg/L and negative TgAb), and TgAb‐positive. A total of 118 patients (146 FNACs) were included: 33 (23%) had negative Tg, 84 (57%) had positive Tg, and 29 (20%) were TgAb‐positive. The positive predictive value (PPV) of neck US was 3%, 50%, and 52% in patients with negative Tg, positive Tg, and positive TgAb, respectively. A sub‐analysis within the Tg‐positive groups using different Tg thresholds revealed PPVs of 29%, 38%, and 58% for Tg concentrations of 0.2 µg/L, < 1.0 µg/L, and ≥ 1.0 µg/L, respectively. The authors concluded that neck US should be reserved for DTC patients with positive Tg (i.e., > 0.2 µg/L) and those with positive TgAb. While the data presented are consistent with previous studies, the observed PPV in patients with Tg levels between 0.2 and 1.0 µg/L appears significantly higher than that reported by Verburg et al. [2]. They evaluated 3176 cervical US exams performed in 773 patients between 1996 and 2012 and found an overall PPV of 23.8% (confidence interval 18.1%–29.5%), with no significant differences between low‐ and high‐risk patients. Notably, no significant differences between patients with undetectable and low detectable (< 1 μg/L) Tg levels were found, contrasting with Seo et al.‘s findings. Several factors may account for these differences. Seo et al. systematically performed FNAC on suspicious findings, whereas Verburg et al. employed a composite outcome. Additionally, Verburg et al. included only patients who underwent radioiodine treatment, whereas Seo et al. also included patients who did not. These methodological differences may partially explain the observed discrepancies. In addition, methodological aspects regarding Tg measurement and interpretation criteria warrant consideration [3].

Firstly, Tg and TgAb immunoassays exhibit significant inter‐assay variability, and different quantitative results are not directly comparable. Van Kinschot et al. tested serum Tg and TgAb in 793 samples derived from 413 patients with DTC using two platforms (Kryptor and Immulite 2000XPi) and found a mean Tg concentration 37.4% lower with the latter. Among 125 samples with positive TgAb in at least one assay, 68 (54.4%) showed discrepancies in TgAb status. Applying guideline‐based cut‐off values for Tg, 33 (4.7%) samples had a Tg concentration ≥ 1.0 μg/L with Immulite and < 1.0 μg/L with Kryptor. Notably, a change in the response to therapy classification occurred in 94 (12.0%) measurements derived from 67 (16.2%) individual patients [4]. Consequently, quantitative differences between laboratories and assays should also be considered when comparing studies.

Secondly, clinical decision limits may differ from analytical limits of quantification and should be established in representative patient series with appropriate statistical approaches [3]. A study including 204 DTC patients was conducted to select the best Tg threshold to predict structural disease after primary treatment, and serum Tg was simultaneously tested with Elecsys and Access immunoassays. Among included patients, 10.8% had structural recurrence and 81.4% showed no evidence of disease at the end of follow‐up. Using receiver operating characteristic (ROC) curve analysis, the best basal Tg cut‐offs to detect structural disease were 0.41 μg/L for Elecsys and 0.36 μg/L for Access assay, respectively. Such thresholds maintained quite an absolute NPV, significantly increasing the PPV compared to functional sensitivities of the two assays (i.e., 0.1 μg/L). Notably, using Cox proportional hazard regression, Tg was the only independent predictor of cancer relapse, and no structural disease occurred in patients with serum Tg below ROC‐derived cutoffs. This study confirmed that clinically oriented statistical analysis (i.e., ROC analysis) allows the definition of assay‐specific clinical decision limits and ameliorates the diagnostic performance [5].

In conclusion, the results presented by Seo et al. confirm the possibility of limiting the use of US in the follow‐up of DTC patients, modulating it based on serum Tg levels. Using a cut‐off close to the limit of quantification of the assay guarantees a very high NPV and reduces the number of required US examinations. In addition, we suggest that the authors 1. report Tg assay(s) employed in their study and report the limit of quantification and/or functional sensitivity of such assay, and 2. apply the ROC analyses to their series to refine the established Tg cut‐off level. We believe this additional data would be very useful for the colleagues involved in the care of patients with DTC.

## Conflicts of Interest

The authors declare no conflicts of interest.
